# Immunohistochemical expression of PAX-8 in Sudanese patients diagnosed with malignant female reproductive tract tumors

**DOI:** 10.1186/s13104-020-05246-4

**Published:** 2020-08-26

**Authors:** Eman T. Ali, Nouh S. Mohamed, Irene R. Shafig, Mohamed S. Muneer, Abubaker Ahmed Yosif, Lamis Ahmed Hassan, Areeg M. Mohamed, Ayman Ahmed, Emmanuel E. Siddig

**Affiliations:** 1 Department of Histopathology and Cytology, Faculty of Medical Laboratory Sciences, University of Khartoum, Khartoum, Sudan; 2 Department of Histopathology and Cytology, Faculty of Medical Laboratory Sciences, National University, Khartoum, Sudan; 3 Alfarrabi College for Science and Technology, Khartoum, Sudan; 4 Faculty of Medicine, Sinnar University, Sennar, Sudan; 5 Molecular Biology Department, Faculty of Medical Laboratory Sciences, Nile University, Khartoum, Sudan; 6 Faculty of Dentistry, Ibn Sina University, Khartoum, Sudan; 7 Department of Neurology, Mayo Clinic, Jacksonville, FL USA; 8 Department of Radiology, Mayo Clinic, Jacksonville, FL USA; 9 Institute of Endemic Diseases, University of Khartoum, Khartoum, Sudan; 10 Mycetoma Research Center, University of Khartoum, Khartoum, Sudan; 11 Faculty of Medicine, Nile University, Khartoum, Sudan

**Keywords:** Female reproductive cancer, Paired box protein-8, Immunohistochemical expression

## Abstract

**Objectives:**

Paired box protein-8 (PAX-8) immunohistochemical expression can be used as a diagnostic marker for epithelial cells tumors. This study aimed at investigating the immunohistochemical expression of PAX-8 among Sudanese females diagnosed with cervical, endometrial, and ovarian cancers between December 2017 and May 2019 by studying their Formalin-fixed paraffin embedded blocks.

**Results:**

Sixty patients diagnosed with female reproductive tract cancers were included who aged 58.7 ± 6.9 years (range, 43—71). Cervix was the most common cancer site in 51/60 (85%) patients. Regarding cancer stage, there was 17 (28%) and 14 (23%) of the study population had stage 3B and 2B, respectively. The histopathological diagnosis included 20 (44%), 13 (29%), and 12 (27%) poorly, moderately, and well differentiated cervical squamous cell carcinoma (SCC) as well as 11 (73%), 2 (13%), 1 (7%), and 1 (7%) endometrial adenocarcinoma, metastatic adenocarcinoma, endocervical adenocarcinoma, and ovarian mucinous cyst adenocarcinoma, respectively. PAX-8 was positively expressed in 9 endometrial adenocarcinoma, 1 endocervical adenocarcinoma and 1 ovarian mucinous cyst adenocarcinoma, 2 poorly, and 1 moderately differentiated SCC. All patients diagnosed with well differentiated SCC and metastatic adenocarcinoma showed no expression of PAX-8. A statistically significant was seen for PAX-8 expression and the different histopathological diagnosis, *P* value < 0.001.

## Introduction

Paired box protein-8 (PAX-8) is a member of the family paired box proteins (PAXs) [1, 2]. PAX-8 consists of 450 amino acids, with a molecular weight of approximately 48 kilo Dalton, and its molecular properties are located on chromosome 2q13 [3–5]. PAX-8 is a transcription factor that regulates organs development during the embryonic period, as well as to maintain normal cellular functions in some cells after birth [6, 7]. During the embryonic period, PAX-8 also plays a significant role in the development of genital organs derived from the mesonephric and the Müllerian ducts [8–10]. In a previous experiment, the deletion of the *PAX-8* gene resulted in dysfunctional uterus, absence of the endometrium, and the vaginal opening. Also, resulted in poor development of the myometrial tissue [11]. Several studies have described the immunohistochemical utility of PAX-8 as a diagnostic marker for epithelial cells neoplasms of many glands and organs such as thyroid, thymus, and kidney as well as some female reproductive tract tumors [12, 13].

In a healthy female reproductive tract, PAX-8 shown to be overexpressed in the epithelial cells of the endocervix and the endometrium [14–16]. PAX-8 was found to be expressed among endometrioid carcinomas, transitional/undifferentiated cell carcinomas, and the metastatic carcinomas at a range, of 38–92%, 67–100%, and [12, 17–23]. Whereas, for the ovarian carcinomas, PAX-8 was under expressed [24]. Considering that, few studies have investigated the immunohistochemical expression of PAX-8 in carcinomas of the endometrium and uterine cervix in the different parts of the world but none from Sudan yet [17, 24–27]. This study aimed at investigating the immunohistochemical expression of PAX-8 among Sudanese females diagnosed with cervical, endometrial, and ovarian carcinomas.

## Main text

### Materials and methods

#### Study design and population characteristics

This is a descriptive, retrospective, hospital- based study conducted at different histopathology laboratories, during the period from December 2017 till May 2019 in Khartoum State, Sudan. We retrieved 60 archived formalin fixed paraffin embedded blocks previously collected from female patients with cervical, endometrial, or ovarian carcinomas. The retrieved formalin fixed paraffin blocks represent all the female population admitted at the hospitals for reproductive malignancies diagnosis. The participants demographic data was collected including age, place of residence. The clinical data including site of cancer, cancer grade, and the histopathological diagnosis were also collected.

#### Sections Preparation for Immunohistochemistry Staining

Two sections were cut using Rotary microtome (Leica, Germany) from each histopathological block. Then, one slide was stained by hematoxylin and eosin staining technique. The other slide was mounted onto 3-aminopropyltriethoxysilane coated slides for immunohistochemistry. To retrieve PAX-8 tissue’s antigen, we treated the sections with citrate buffer at 96° C for 10 min in a water-bath. Then, the tissue sections were rinsed first in distilled water and later with Tris buffer saline (TBS). This was followed by treatment with peroxidase block (3% hydrogen peroxide in methyl alcohol) for 15 min to quench endogenous peroxidase activity. The slides were then placed in a humid chamber. Then, the slides were drained and rinsed in two successive changes of Tris buffer (wash buffer) for 5 min each. Nonspecific protein–protein interactions were blocked by incubating and treating the tissue sections in a humid chamber with the power block (casein in phosphate buffered saline) for 10 min. Then, the remaining solution was drained from the slides. The sections were then incubated in the primary antibody PAX-8; anti-PAX-8 rabbit anti-human monoclonal antibody (ab189249; Abcam, United Kingdom) at room temperature in the humid chamber according to the manufacture instructions.

Observing the yellowish-brown or brown appearance of the nucleus was considered a positive result for the PAX-8. For the negative control, we omitted the incubation with the primary antibody step; instead we incubated the section in the phosphate buffer saline (PBS).

#### Results interpretations

For the interpretation of the results we depended on the intensity as well as the number of the cells that expressed the marker and the expression was graded into 5 categories: Negative: No staining; 1 +  = less than 25% of the cells were expressing the marker; 2 +  = 25–50% of the cells were expressing the marker; 3 +  = more than 50–75% of the cells were expressing the marker; 4 +  = more than 75% of the cells were expressing the marker. The slides were interpreted and validated by two expert pathologists, blindly of each other results. Photomicrographs were taken using Olympus SP-350 camera (Olympus Imaging America Inc., USA).

#### Statistical analysis

The statistical analysis of the results was done using IBM SPSS Statistics (vs. 16.0). The Chi-Squared test was performed to compare the frequencies of categorical variables. Statistical significance level was defined as *p* value < 0.05 at 95% confidence interval.

### Results

#### Characteristics of the study participants

The study included 60 patients diagnosed with female genital tract cancer. Patients aged 58.7 ± 6.9 years (range 43–71 years). Patients were grouped into 4 age groups. Those aged 50–59 years constituted half of the study participants; 30/60 (50.0%). The remaining were 11/60 (18.3%), 16/60 (26.7%), and 3/60 (5.0%) patients distributed across the remaining age groups of 40–49 years, 60–69 years, and 70–79 years, respectively. According to patients’ place of residence, patients were originating from the four regions of Sudan. Most of the patients, 27/60 (45.0%), were from western part of Sudan followed by 19/60 (32.0%) from the central part of Sudan.

Regarding the site of cancer, the cervix was the most commonly involved; 51/60 (85.0%) patients. There were 5/60 (8.3%) and 4/60 (6.7%); endometrial and ovarian cancer, respectively. Based on the International Federation of Gynecology and Obstetrics (FIGO) cancer grading, the majority of the study population was diagnosed with stage 3B and 2B cancer; 17/60 (28.3%) and 14/60 (23.3%) of the patients, respectively. The were 9/60 (15.0%), 8/60 (13.3%), 6/60 (10.0%), 3/60 (5.0%), and 3/60 (5.0%) stage 4B, 3A, 2A, 1B, and 4A, respectively. No statistically significant association between FIGO staging and age group was found (*P* value 0.279).

Histologically, there were 45/60 (75.0%) squamous cell carcinoma (SCC): all of which were cervical cancers; and 15/60 (25.0%) adenocarcinoma. SCC and adenocarcinoma were further classified into 20/60 (33.3%) poorly differentiated SCC, 13/60 (21.7%) moderately differentiated SCC, and 12/60 (20.0%) well differentiated SCC, 11/60 (18.3%) endometrium adenocarcinoma, 2/60 (3.3%) metastatic adenocarcinoma, 1/60 (1.7%) endocervical adenocarcinoma, and 1/60 (1.7%) ovarian mucinous cyst adenocarcinoma.

Based on age groups, age group showed no statistically significant relationship with either patients’ place of residence, cancer site, cancer histological type, FIGO staging, and cancer histopathological type (Table 1).

**Table 1:** Classification of Participants demographic and clinical diagnosis based on age group

	Age group no. (%)				Total no. 60	*P* value
	40—49 years	50—59 years	60—69 years	70—79 years		
Residence of patient						
Central Sudan	2 (10.5)	12 (63.2)	4 (21.1)	1 (5.3)	19 (31.7)	0.550
East Sudan	1 (16.7)	5 (83.3)	0 (0.0)	0 (0.0)	6 (10.0)	
West Sudan	6 (22.2)	10 (37.0)	9 (33.3)	2 (7.4)	27 (45.0)	
North Sudan	2 (25.0)	3 (37.5)	3 (37.5)	0 (0.0)	8 (13.3)	
Site of cancer						
Cervix	10 (19.6)	25 (49.0)	13 (25.5)	3 (5.9)	51 (85.0)	0.885
Endometrium	1 (20.0)	3 (60.0)	1 (20.0)	0 (0.0)	5 (8.3)	
Ovary	0 (0.0)	2 (40.0)	2 (40.0)	0 (0.0)	4 (6.7)	
Cancer histological type						
SCC	9 (20.0)	22 (48.9)	13 (28.9)	1 (2.2)	45 (75.0)	0.330
Adenocarcinoma	2 (13.0)	8 (53.3)	3 (20.0)	2 (13.3)	15 (25.0)	
FIGO staging						
Stage 1	0 (0.0)	2 (66.7)	1 (33.3)	0 (0.0)	3 (5.0)	0.279
Stage 2A	1 (16.7)	2 (33.3)	3 (50.0)	0 (0.0)	6 (10.0)	
Stage 2B	5 (35.7)	7 (50.0)	2 (14.3)	0 (0.0)	14 (23.3)	
Stage 3A	1 (12.5)	4 (50.0)	3 (37.5)	0 (0.0)	8 (13.3)	
Stage 3B	2 (11.8)	11 (64.7)	2 (11.8)	2 (11.8)	17 (28.3)	
Stage 4A	0 (0.0)	0 (0.0)	2 (66.7)	1 (33.3)	3 (5.0)	
Stage 4B	2 (22.2)	4 (44.4)	3 (33.3)	0 (0.0)	9 (15.0)	
Histopathological cancer grades						
Well differentiated SCC	2 (16.7)	5 (41.7)	5 (41.7)	0 (0.0)	12 (20.0)	0.633
Poorly differentiated SCC	3 (15.0)	10 (50.0)	6 (30.0)	1 (5.0)	20 (33.3)	
Moderately differentiated SCC	4 (30.8)	7 (53.8)	2 (15.4)	0 (0.0)	13 (21.7)	
Endometrium adenocarcinoma	1 (9.1)	6 (54.5)	2 (18.2)	2 (18.2)	11 (18.3)	
Endocervical adenocarcinoma	1 (100)	0 (0.0)	0 (0.0)	0 (0.0)	1 (1.7)	
Metastatic adenocarcinoma	0 (0.0)	1 (50.0)	1 (50.0)	0 (0.0)	2 (3.3)	
Ovarian mucinous cyst adenocarcinoma	0 (0.0)	1 (100)	0 (0.0)	0 (0.0)	1 (1.7)	

*SCC* Squamous Cell Carcinoma

#### Immunohistochemical Expression of PAX-8

**Fig. 1:**
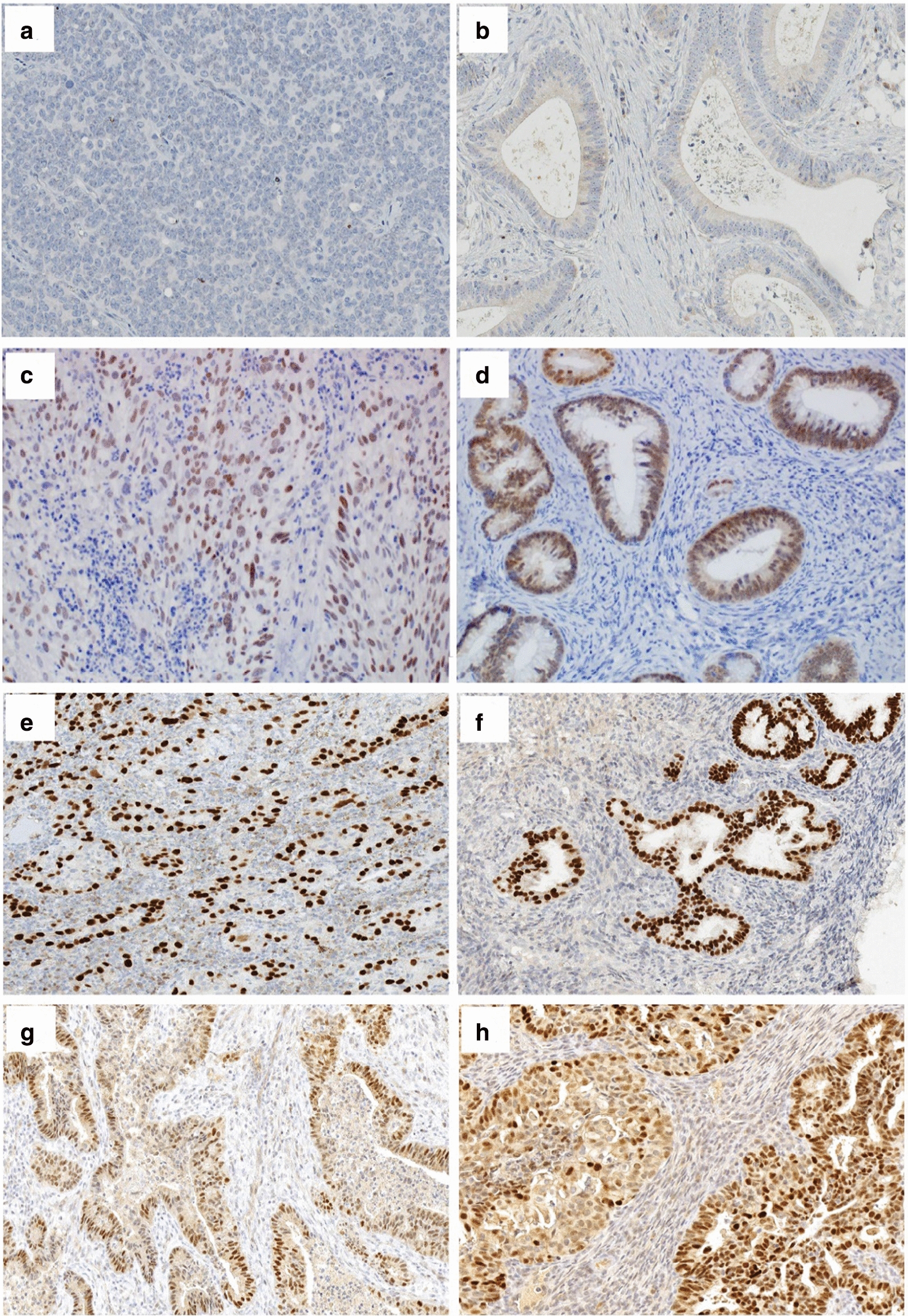
Immunohistochemical expression of PAX8 among the different histopathological cancer types and grades. The immunohistochemical expression of PAX-8 is shown as a yellowish-brown or brown staining of the nucleus. **a** Well differentiated SCC (negative); **b** Metastatic adenocarcinoma (negative). **c **Poorly differentiated SCC (positive); **d** Moderately differentiated SCC (positive); **e** Endometrium adenocarcinoma (positive); **f** Ovarian mucinous cystadenocarcinoma (positive); **g** Endocervical adenocarcinoma (positive); and **h** endometroid adenocarcinoma (positive)

The immunohistochemical expression of PAX-8 was shown as a yellowish-brown or brown staining of the nucleus (Fig. 1). Based on site of cancer, all endometrium carcinoma showed positive expression of PAX-8 with *P* value < 0.001. There were only 14 patients who had positive expression of PAX-8; including 11/15 (73.3%) adenocarcinoma and 3/45 (6.7%) SCC. A statistically significant difference was noted for the PAX-8 staining and cancer type with *P* value < 0.001. The analysis of PAX-8 staining results based on the histopathological diagnosis showed that all patients who were diagnosed with well differentiated SCC and metastatic adenocarcinoma had negative results for the PAX-8 expression. While 9/11 (81.8%) of the endometrium adenocarcinoma were found positive for the PAX-8 expression. A statistically significan was t seen for PAX-8 expression and the different histopathological diagnosis, *P* value < 0.001 (Table 2).

**Table 2:** Association of clinical diagnosis and the immunohistochemical expression of PAX-8

	PAX 8 results no. (%)		Total no. 60	*P* value
	Positive	Negative		
Cancer histological type				
SCC	3 (6.7)	42 (93.3)	45 (75.0)	< 0.001
Adenocarcinoma	11 (73.3)	4 (26.7)	15 (25.0)	
Cancer site				
Cervix	8 (15.7)	43 (84.3)	51 (85.0)	< 0.001
Endometrium	5 (100)	0 (0.0)	5 (8.3)	
Ovary	1 (25.0)	3 (75.0)	4 (6.7)	
FIGO staging				
Stage 1	1 (33.3)	2 (66.7)	3 (5.0)	0.034
Stage 2A	0 (0.0)	6 (100)	6 (10.0)	
Stage 2B	3 (21.4)	11 (78.6)	14 (23.3)	
Stage 3A	2 (25.0)	6 (75.0)	8 (13.3)	
Stage 3B	2 (11.8)	15 (88.2)	17 (28.3)	
Stage 4A	0 (0.0)	3 (100)	3 (5.0)	
Stage 4B	6 (66.7)	3 (33.3)	9 (15.0)	
Cancer histopathological grading				
Well differentiated SCC	0 (0.0)	12 (100)	12 (20.0)	< 0.001
Poorly differentiated SCC	2 (10.0)	18 (90.0)	20 (33.3)	
Moderately differentiated SCC	1 (7.7)	12 (92.3)	13 (21.7)	
Endometrium adenocarcinoma	9 (81.8)	2 (18.2)	11 (18.3)	
Endocervical adenocarcinoma	1 (100)	0 (0.0)	1 (1.7)	
Metastatic adenocarcinoma	0 (0.0)	2 (100)	2 (3.3)	
Ovarian mucinous cyst adenocarcinoma	1 (100)	0 (0.0)	1 (1.7)	

*SCC* Squamous Cell Carcinoma

### Discussion

Previous studies on the immunohistochemical expression of PAX-8 in the normal female reproductive tract showed that PAX-8 was expressed in the endometrial, endocervical, and ovarian epithelial cells, as well as in non-ciliated epithelial cells of the fallopian tubes [17, 20, 28, 29]. This study investigated the immunohistochemical expression of PAX-8 in Sudanese patients who were diagnosed with female reproductive tract cancers. Patients on the 5th decade of life were constituting half of the study participants with no statistically significant association between age group and the type of cancer. However, previous studies had suggested other risk factors which could contribute in the development of certain gynecological cancer [30, 31].

Regarding the place of residence, the majority of patients coming from western Sudan. This result is in contrary with a previous study in Sudan conducted by Saeed et al. in which they showed that the percentage of patients suffering from different types of cancers residing in central and northern Sudan were higher compared to the other regions in Sudan [32]. Nevertheless, these findings could suggest the involvement of environmental risk factors; however, the limited study samples size is insufficient to support this suggestion. Therefore, further research with a larger samples size investigating the potential environmental risk factors is essential for strategic prevention and protection measures.

The reported number of female patients with cervical cancer was high compared to ovarian and endometrium cancer. Similar results were seen previously among Sudanese females [32]. Also, the high frequency of stages 3B and 2B compared to the other stages were comparable to previous study conducted in Sudan [33]. This similarity underscores a delayed response among Sudanese females in seeking healthcare, and urge the need for health promotion and education to encourage young Sudanese females for the early signs, detection, and seeking healthcare as early as possible for a better treatment.

Regarding the classification based on the histopathological diagnosis, most of the female diagnosed with SCC. This result was also similar to previous study investigated the prevalence of the different gynecologic cancer in Sudan [34]. However, the expression of PAX-8 among the studied samples was relatively low compared to previous studies [17, 24, 28, 35, 36], this could be attributed to the site of cancer development. While agrees with another study, where PAX-8 was expressed only in 1/60 patient [35].

Interestingly, a high frequency of PAX-8 expression was noted among females diagnosed with endometrium cancer compared to SCC, this finding is in contrary with a previous report where PAX-8 was expressed among only 3% of the studied samples [37]. Also, the result was strongly in accordance with other studies [13, 38, 39]. Besides that, the lack of PAX-8 expression among those who were diagnosed with well differentiated SCC and metastatic adenocarcinoma could play a significant role in either gynecologic cancer differentiation or in detection of endometrium adenocarcinoma progression to metastatic adenocarcinoma [40, 41].

### Conclusion

Although PAX-8 showed a significant expression among adenocarcinomas lesions and negative expression was noted among those with well differentiated SCC and metastatic adenocarcinoma, PAX-8 might not be beneficial when used alone as a diagnostic marker for the tumors that occur in the female reproductive tract.

## Limitations

- The small sample size investigated in this study reduced the ability of using the expression of PAX-8 as a diagnostic marker. Therefore, a large-scale study is needed and it should include other types of malignant tumors encountered in the female reproductive system.

## Data Availability

The datasets used and/or analyzed during the current study are available from the corresponding author on reasonable request.
